# Intra-Aortic Balloon Pump Counterpulsation during Primary Percutaneous Coronary Intervention for ST-Elevation Myocardial Infarction and Cardiogenic Shock: Insights from the British Columbia Cardiac Registry

**DOI:** 10.1371/journal.pone.0148931

**Published:** 2016-02-12

**Authors:** M. Bilal Iqbal, Simon D. Robinson, Lillian Ding, Anthony Fung, Eve Aymong, Albert W. Chan, Steven Hodge, Anthony Della Siega, Imad J. Nadra, British Columbia Cardiac Registry Investigators

**Affiliations:** 1 Department of Cardiology, Victoria Heart Institute Foundation, Victoria, BC, Canada; 2 Department of Cardiology, Royal Jubilee Hospital, Victoria, BC, Canada; 3 Cardiac Services BC, Provincial Health Services Authority, Vancouver, BC, Canada; 4 Department of Cardiology, Vancouver General Hospital, Vancouver, BC, Canada; 5 Department of Cardiology, St. Paul's Hospital, Vancouver, BC, Canada; 6 Department of Cardiology, Royal Columbian Hospital, Vancouver, BC, Canada; 7 Department of Cardiology, Kelowna General Hospital, Kelowna, BC, Canada; Medizinische Hochschule Hannover, GERMANY

## Abstract

**Background:**

Cardiogenic shock complicating ST-elevation myocardial infarction (STEMI) is associated with significant morbidity and mortality. In the primary percutaneous coronary intervention (PPCI) era, randomized trials have not shown a survival benefit with intra-aortic balloon pump (IABP) therapy. This differs to observational data which show a detrimental effect, potentially reflecting bias and confounding. Without robust and valid risk adjustment, findings from non-randomized studies may remain biased.

**Methods:**

We compared long-term mortality following IABP therapy in patients with cardiogenic shock undergoing PPCI during 2008–2013 from the British Columbia Cardiac Registry. We addressed measured and unmeasured confounding using propensity score and instrumental variable methods.

**Results:**

A total of 12,105 patients with STEMI were treated with PPCI during the study period. Of these, 700 patients (5.8%) had cardiogenic shock. Of the patients with cardiogenic shock, 255 patients (36%) received IABP therapy. Multivariable analyses identified IABP therapy to be associated with increased mortality up to 3 years (HR = 1.67, 95% CI:1.20–2.67, p<0.001). This association was lost in propensity-matched analyses (HR = 1.23, 95% CI: 0.84–1.80, p = 0.288). When addressing measured and unmeasured confounders, instrumental variable analyses demonstrated that IABP therapy was not associated with mortality at 3 years (Δ = 16.7%, 95% CI: -12.7%, 46.1%, p = 0.281). Subgroup analyses demonstrated IABP was associated with increased mortality in non-diabetics; patients not undergoing multivessel intervention; patients without renal disease and patients not having received prior thrombolysis.

**Conclusions:**

In this observational analysis of patients with STEMI and cardiogenic shock, when adjusting for confounding, IABP therapy had a neutral effect with no association with long-term mortality. These findings differ to previously reported observational studies, but are in keeping with randomized trial data.

## Introduction

Cardiogenic shock complicating acute ST-elevation myocardial infarction (STEMI) is associated with significant morbidity and mortality[1, 2]. Early revascularisation therapy has been shown to improve outcomes in these patients[1]. Despite the use of primary percutaneous coronary intervention (PPCI), mortality remains high[3]. The intra-aortic balloon pump (IABP) is the most widely used mechanical hemodynamic support device in acute cardiac care[4]. It improves diastolic blood pressure, thereby augmenting coronary perfusion and its afterload reduction properties reduce myocardial oxygen consumption and increase cardiac output[5]. These physiological principles would suggest that IABP therapy might improve outcomes for patients with cardiogenic shock. In the PPCI era, observational studies have shown that IABP therapy is associated with increased mortality[6], whilst randomized trial data have shown a neutral effect[7]. This discrepancy may reflect bias and confounding inherent to observational studies and even with traditional methods of adjustment, residual confounding is expected[8]. We conducted an observational analysis to explore the relationship between IABP therapy and long-term mortality in patients receiving PPCI for STEMI with cardiogenic shock and utilized statistical approaches to address measured and unmeasured confounding.

## Methods

This was a retrospective observational cohort study to investigate the relationship between long-term survival and IABP use at the time of PPCI for patients presenting with STEMI and cardiogenic shock. We used merged datasets from the British Columbia Cardiac Registry.

### British Columbia Cardiac Registry

The British Columbia Cardiac Registry (BCCR) collects demographic, clinical and procedural data on all patients who undergo invasive cardiac procedures in British Columbia. Data is collected prospectively from five tertiary cardiac centers and entered into a central database maintained by a dedicated management team. Mortality events are obtained by linkage of provincial Vital Statistics Database and are automatically entered into the BCCR which records the date of death for all patients.

### Population study and design

We examined an observational cohort of consecutive patients treated with PPCI between 2008–2013 at all 5 cardiac centers in British Columbia, Canada. Patient and procedural details were recorded at the time of the procedure. Anonymous datasets with linked mortality data were used for analysis. Initially, we identified 12,105 patients with STEMI that were treated with PPCI. The BCCR only records cardiogenic shock status at the time of PCI procedure. Data on subsequent development of cardiogenic shock post-procedure during the hospital admission is not captured by the database. Of the patients with STEMI undergoing PPCI, 700 patients (5.8%) had cardiogenic shock at the time of the procedure and were therefore included in the final analysis.

### Definitions and clinical outcomes

Cardiogenic shock was defined as a sustained (>30 minutes) episode of systolic blood pressure <90 mm Hg secondary to cardiac dysfunction, and/or the requirement for inotropic or mechanical support to maintain blood pressure and adequate systemic perfusion[9]. We analyzed all-cause mortality at 30 days, 1 year and 3 years.

### Ethics

All patient identifiable information was removed prior to database merging and analysis. Ethical approval for this study was obtained from the University of British Columbia Ethics Board (H12-01628).

### Statistical analyses

Patients were divided into "no IABP" and "IABP" groups. Non-categorical variables in our dataset had a skewed distribution, and thus were summarized using median (lower and upper quartiles) and compared using the Mann-Whitney U-test. Categorical variables were expressed as percentages and compared using the Z-test. All statistical analyses were performed using MedCalc v12.5 (MedCalc Software, Ostend, Belgium) and R (Foundation for Statistical Computing, Vienna, Austria). Statistical significance was established at p<0.05 (2-tailed) for all tests.

#### (a) Multivariable-adjusted models for mortality

To determine independent predictors for mortality, Cox proportional hazards regression models were used to provide adjusted hazard ratios (HRs) with 95% confidence intervals (CIs). The proportional hazards assumption was tested and verified with Schoenfeld residuals. To guide selection of significant variables for the final multivariable model, we initially adjusted for age, sex, diabetes, GP2b-3a inhibitor use, previous MI, previous history of revascularization, hypertension, hypercholesterolemia, renal disease, previous cerebrovascular accident, peripheral vascular disease, smoking, history of heart failure, radial access, vessel intervened upon (LMS, LAD, LCx, RCA, graft), pre-procedural thrombolysis, DES use, aspiration thrombectomy, severe LV dysfunction, multivessel disease, multivessel intervention, pulmonary disease, liver/gastrointestinal disease and malignancy using a stepwise variable selection process. The significant covariates from this were then included in the final multivariate model with IABP as a forced-in variable. Thus the covariates used in the final multivariate model were age, female sex, diabetes, renal disease, pre-procedural thrombolysis, LMS intervention, proximal LAD intervention, multivessel intervention, GP2b-3a inhibitor use and IABP use. In this way, the number of variables was limited to 1 per ≥ 10 events to prevent over-fitting of the model. Cumulative mortality rates were also presented as Kaplan-Meier curves and compared with the log-rank test.

#### (b) Propensity Score Matching

To account for measured confounders, propensity matching was performed. To derive propensity scores (*PS*_*i*_), a logistic regression model was fitted for IABP therapy to patient demographics, clinical, anatomical and procedural variables. Propensity score matching was performed using nearest-neighbor matching and 1:1 matching without replacement using calipers set at 0.1[10]. Cumulative mortality rates were also presented as Kaplan-Meier curves and compared with a stratified log-rank test. The propensity score models were assessed using the receiver operator curve (ROC) analysis (c-statistic) and Hosmer-Lemeshow test. Covariate balance was assessed using absolute standardised differences in means for the propensity-matched cohorts, with differences less than 10% taken to indicate good balance[10]. Cox proportional hazards regression models and logistic regression models were then applied to the propensity-matched cohorts adjusting for the significant covariates identified from multivariate models and *PS*_*i*_ (double-robust models)[11].

#### (c) Instrumental variable (IV) analysis

IV analysis is an econometric method used to remove the effects of hidden bias in observational studies[12]. An IV has 2 key characteristics: (a) it is highly correlated with the treatment and (b) does not independently affect the outcome, other than via its effects through the treatment, so that it is not associated with measured or unmeasured variables. The geographical treatment rate can serve as effective IV[8] and we demonstrated this to be the case with centre-specific IABP rate of use (low vs. high). The rate of IABP use was determined for each centre, and each center was classified as either as having "high" or "low" center -specific IABP rate of use depending on whether it was greater or smaller than the median rate of use. We initially performed unadjusted and adjusted linear regression, adjusting for the same covariates as in the Cox proportional hazards models (as above). An adjusted IV analysis was performed using a simultaneous 2-stage least-squares regression approach. Finally, we adapted the theoretical framework proposed by Brookhart and Schneeweiss[13] to examine the strength of the IV across various patient subgroups. Adopting this framework, IABP use was determined for low vs. high centre IABP rates for each covariate. If the variation in IABP use induced by the IV for each covariate is larger or smaller than that observed in the overall cohort, it is possible that variation across unmeasured factors may bias the estimates for the effect of IABP in the population under study.

## Results

### Baseline population and procedural characteristics

**Fig 1:**
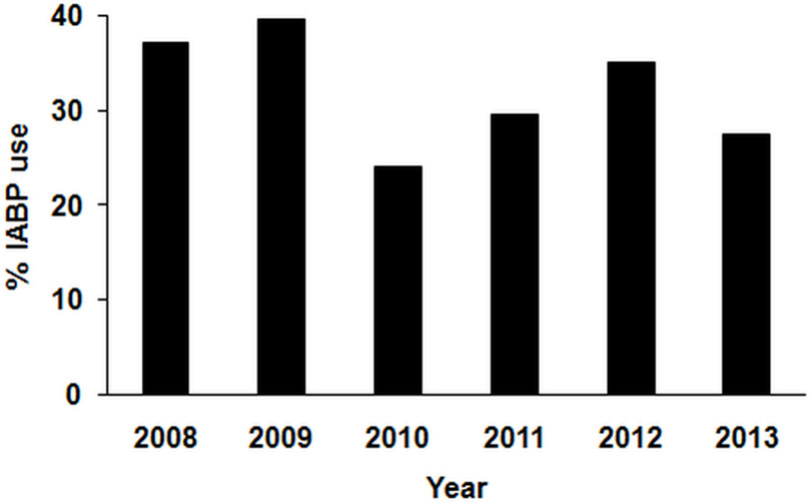
Trends in IABP use over the study period (2008–2013).

We analyzed 700 consecutive patients who underwent PPCI for STEMI with cardiogenic shock across all 5 tertiary cardiac centers in British Columbia. Of this patient population, 225 patients received IABP therapy (32%) and 475 patients did not receive IABP therapy (68%). The trends in IABP use over the study period are shown in Fig 1. Patients receiving IABP therapy were more likely to have renal disease; have previous revascularization; have severe LV dysfunction; have multivessel disease; receive GP 2b-3a inhibitor; undergo transfemoral intervention; receive LMS/LAD intervention; undergo multivessel intervention; and have poor pre- and post procedural TIMI flow. The patient characteristics are summarized in Table 1.

**Table 1:** Baseline demographic and procedural characteristics for total study population.

	Total (n = 700)	No IABP (n = 475)	IABP (n = 255)	p value
**Clinical factors**				
Age (years)	66(56,75)	65(56,75)	66(56,75)	0.732
Female	25.7	26.7	23.6	0.368
PVD	6.7	7.2	5.6	0.454
Renal disease *	12.1	9.3	18.0	0.002
Previous CVA	7.5	7.0	8.5	0.487
Previous MI	17.0	15.6	20.0	0.146
Previous revascularization	19.3	16.8	25.4	0.012
History of heart failure	16.0	14.6	19.0	0.148
Diabetes	24.2	23.1	26.6	0.315
Hypertension	48.9	49.0	48.8	0.969
Hypercholesterolemia	34.9	36.0	32.5	0.386
Smoking ^†^	28.3	32.0	20.6	0.003
Severe LV (EF<30%)	23.1	12.7	47.6	<0.001
Pulmonary disease	12.0	12.2	11.5	0.811
Gastrointestinal disease	10.2	10.4	9.7	0.776
Malignancy	6.5	6.8	5.8	0.643
**Coronary anatomy** ^§^				
LMS	9.0	5.9	15.7	<0.001
LAD	77.4	72.6	87.4	<0.001
proximal LAD	39.4	34.1	50.7	<0.001
non-proximal LAD	62.9	56.6	76.2	<0.001
LCx	57.9	52.4	69.5	<0.001
RCA	69.3	67.6	73.1	0.141
Multivessel disease	70.8	65.3	82.5	<0.001
**Procedural characteristics**				
Pre-procedural thrombolysis	14.6	17.5	8.4	0.002
Radial access	23.9	33.7	3.1	<0.001
GP 2b/3a inhibitor use	25.0	21.9	31.6	0.005
Thrombectomy	30.1	32.2	25.8	0.083
DES use	45.0	46.0	43.1	0.505
Pre-procedural TIMI flow				
TIMI 0–1	95.1	93.0	99.5	<0.001
TIMI 2–3	4.9	7.0	0.5	<0.001
Post-procedural TIMI flow				
TIMI 0–1	90.1	85.7	99.5	<0.001
TIMI 2–3	9.9	14.3	0.5	<0.001
**Target vessel**				
LMS	6.6	3.4	13.5	<0.001
LAD	48.1	42.7	59.6	<0.001
proximal LAD	29.7	23.4	43.0	<0.001
non-proximal LAD	33.4	30.1	40.4	<0.001
LCx	27.1	21.5	39.0	<0.001
RCA	43.7	46.9	36.8	0.011
Graft	1.1	1.3	0.9	0.672
Multivessel intervention	22.8	15.7	37.6	<0.001

Discrete variables are presented as percentages and compared using the Z-test (2-tailed); Continuous data presented as medians (25% IQ, 75% IQ) and compared using the Mann-Whitney U-test (2-tailed).

* Renal disease was defined as serum creatinine >150mmol/l or renal replacement therapy.

^†^ Smoking was defined as smoking of ≥1 cigarettes/day and had smoked in the month preceding PCI

^§^ A diseased epicardial coronary vessel was defined as having a >50% coronary stenosis by visual estimation.

Abbreviations: CVA, cerebrovascular accident; MI, myocardial infarction; LMS, left main-stem artery; LAD, left anterior descending artery; LCx, left circumflex artery; RCA, right coronary artery; IABP, intra-aortic balloon pump; and DES, drug-eluting stent.

### Unadjusted mortality

The use of IABP was associated with higher mortality at 30 days (38.2% vs. 17.9%, p<0.001); 1 year (44.9% vs. 22.7%, p<0.001); and 3 years (47.1% vs. 26.3%, p<0.001).

### Cox proportional hazards regression models for mortality

When adjusting for baseline clinical, anatomical and procedural variables, multivariable-adjusted analyses identified IABP use as an independent predictor for mortality at 30 days (HR = 2.02, 95% CI:1.40–2.91, p<0.001); 1 year (HR = 1.87, 95% CI:1.34–2.61, p<0.001); and 3 years (HR = 1.67, 95% CI:1.20–2.67, p<0.001).

### Propensity matched analyses

**Fig 2:**
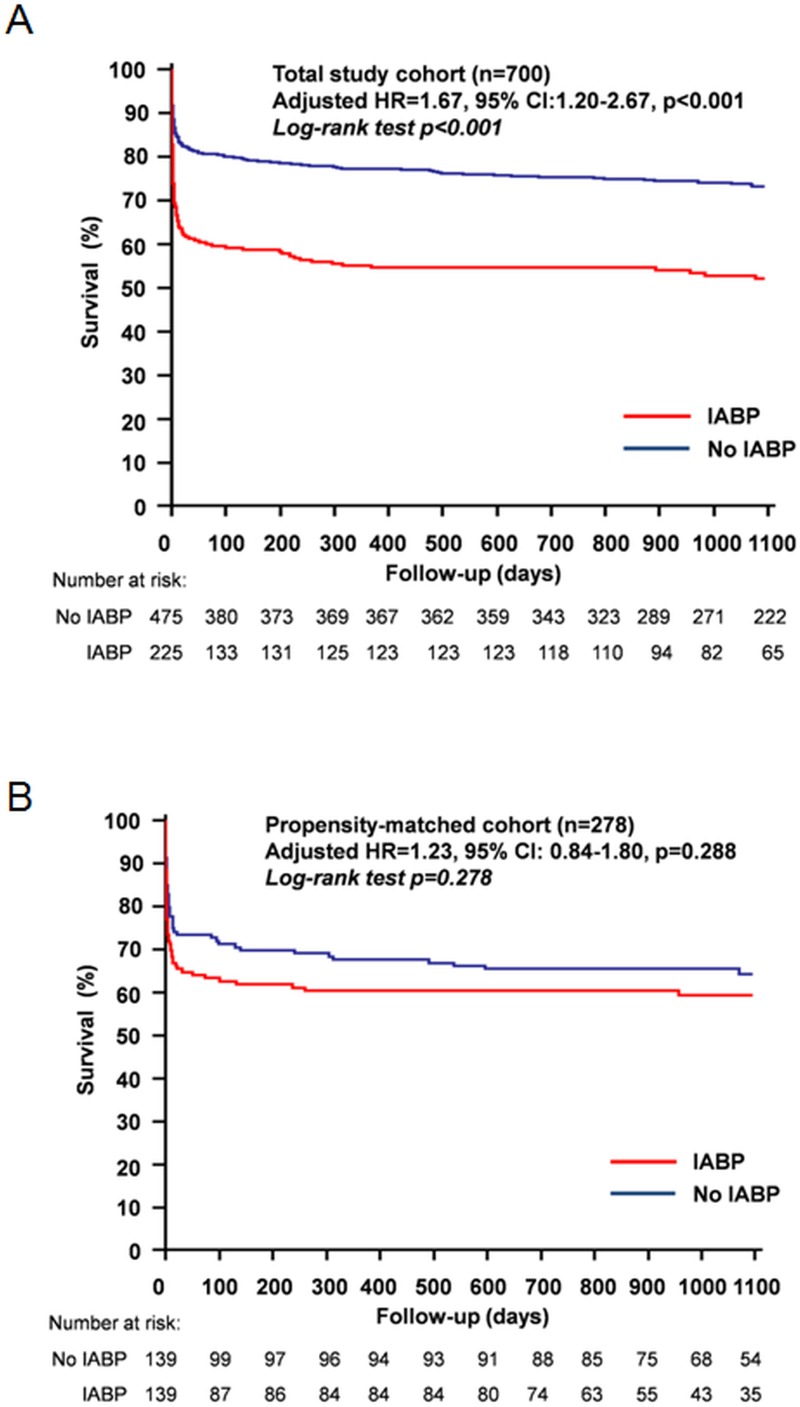
Kaplan-Meier survival curves at 3 years. (A) unmatched cohort (n = 700). (B) propensity-matched cohort (n = 278).

Propensity score (PS_i_) matching yielded a total of 278 matched patients (139 patients in each group). The c-statistics for the PS_i_ model was 0.86 and Hosmer-Lemeshow test yielded a p = 0.651. Table 2 illustrates that the baseline demographics, clinical, anatomical and procedural variables were well balanced in the propensity-matched cohorts and the absolute standardized differences were all less than 10% (Fig 2). IABP use demonstrated a non-significant trend towards higher mortality at 30 days (34.5% vs. 26.6%, p = 0.152); but was not associated with higher mortality at 1 year (39.6% vs. 32.4%, p = 0.211); and 3 years (40.3% vs. 35.3%, p = 0.387). Multivariable-adjusted models demonstrated that IABP use was not associated with mortality at 30 days (HR = 1.37, 95% CI: 0.89–2.11, p = 0.146), 1 year (HR = 1.31, 95% CI: 0.89–1.94, p = 0.177); and 3 years (HR = 1.23, 95% CI: 0.84–1.80, p = 0.288). As a secondary analysis, when we performed propensity matching using a 1: many matching to utilize a greater patient pool (n = 348), IABP use was still not associated with mortality at 3 years (HR = 1.28, 95% CI: 0.89–1.82, p = 0.138).

**Table 2:** Distribution of covariates in the propensity-matched cohorts.

	Total (n = 278)	No IABP (n = 139)	IABP (n = 139)	p value	Sdiff
**Clinical factors**					
Age > 80 years	18.7	19.4	18.0	0.758	3.7
Female	27.7	28.1	27.3	0.893	1.6
PVD	5.0	4.6	5.5	0.754	3.9
Renal disease*	15.5	15.7	15.4	0.946	0.9
Previous CVA	10.1	10.8	9.4	0.710	4.6
Previous MI	19.1	19.4	18.7	0.879	1.8
Previous revascularization	21.6	21.6	21.6	1.000	0.0
History of heart failure	18.1	18.6	17.6	0.835	2.6
Diabetes	26.5	25.2	27.9	0.677	6.2
Hypertension	47.7	45.7	49.6	0.535	7.8
Hypercholesterolemia	31.1	31.3	31.0	0.959	0.6
Smoking ^†^	24.3	25.6	23.0	0.633	6.0
Severe LV (EF<30%)	27.6	27.8	27.4	0.950	0.9
Pulmonary disease	14.6	15.6	13.5	0.630	6.1
Gastrointestinal disease	9.5	9.4	9.6	0.967	0.5
Malignancy	6.7	7.0	6.4	0.854	2.3
**Coronary anatomy** ^§^					
LMS	10.4	10.1	10.8	0.844	2.4
LAD	80.6	79.1	82.0	0.544	7.3
proximal LAD	41.4	39.6	43.2	0.543	7.3
non-proximal LAD	70.1	69.1	71.2	0.694	4.7
LCx	60.8	59.0	62.6	0.539	7.4
RCA	70.5	70.5	70.5	1.000	0.0
Multivessel disease	75.2	75.5	74.8	0.890	1.7
**Procedural characteristics**					
Pre-procedural thrombolysis	10.1	10.1	10.1	1.000	0.0
Radial access	5.4	5.8	5.0	0.791	3.2
GP 2b/3a inhibitor use	27.7	27.3	28.1	0.893	1.6
Thrombectomy	28.8	27.3	30.2	0.596	6.4
DES use	44.4	44.3	44.4	0.806	0.4
Post-procedural TIMI flow					
TIMI 0–1	99.2	99.2	99.2	0.982	0.3
TIMI 2–3	0.8	0.8	0.8	0.982	0.3
**Target vessel**					
LMS	6.8	7.2	6.5	0.812	2.9
LAD	47.1	46.0	48.2	0.719	4.3
proximal LAD	30.6	30.2	30.9	0.896	1.6
non-proximal LAD	34.5	35.3	33.8	0.801	3.0
LCx	31.7	33.1	30.2	0.606	6.2
RCA	41.4	41.7	41.0	0.903	1.5
Graft	1.4	1.4	1.4	1.000	0.0
Multivessel intervention	26.7	28.1	25.4	0.587	6.1

Discrete variables are presented as percentages and compared using the Z-test (2-tailed); Continuous data presented as medians (25% IQ, 75% IQ) and compared using the Mann-Whitney U-test (2-tailed). All variables were also compared using absolute standardized difference in means (%) (S_diff_).

* Renal disease was defined as serum creatinine >150mmol/l or renal replacement therapy.

^†^ Smoking was defined as smoking of ≥1 cigarettes/day and had smoked in the month preceding PCI

^§^ A diseased epicardial coronary vessel was defined as having a >50% coronary stenosis by visual estimation.

Abbreviations are as for Table 1.

### Kaplan-Meier survival analyses

The Kaplan-Meier survival curves for the unmatched and propensity-matched cohorts are shown in Fig 2. When adjusting for measured confounding, there was no difference in long-term survival with IABP therapy (stratified log rank test p = 0.278).

### Subgroup analyses

**Fig 3:**
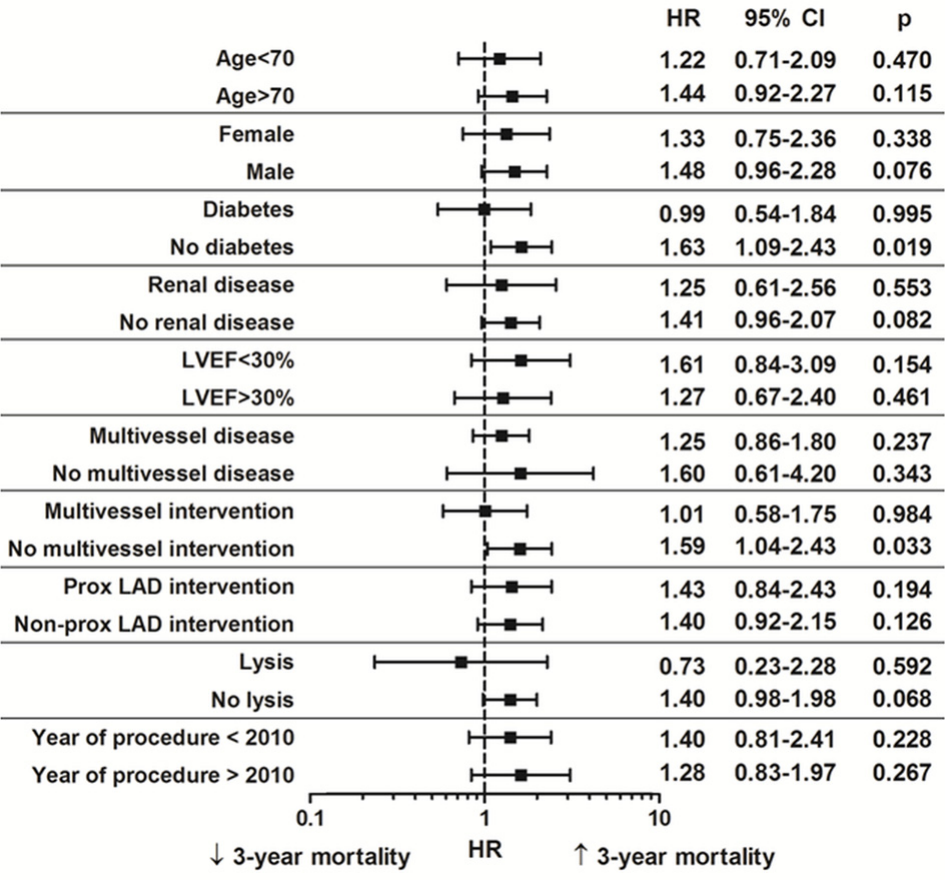
Subgroup analyses in select patient groups. Multivariable adjusted models for 3 year mortality referent to IABP therapy. There were no significant interactions by subgroups.

We performed subgroup analyses incorporating PS_i_ as a covariate into the multivariable models[14]. This method allowed us to perform subgroup analyses where residual confounding was present despite standard adjustment methods. The results of the subgroup analyses are shown in Fig 3.

### Instrumental variable analyses

To account for unmeasured confounding, IV analysis was performed using centre-specific IABP rate of use (low vs. high) as an IV. The use of IABP was 12.8% vs. 42.5% (low vs. high groups, p<0.001), and the strong association between IABP use and the IV was validated using logistic and linear regression models. Multivariable models demonstrated that the IV was not associated with mortality and the F-test statistic for the IV was 70.81 (value <10 indicates a weak IV). Table 3 demonstrates the strength of centre-specific IABP rate as an IV. When comparing baseline variables, although there were differences in covariates between the groups, these differences were smaller and the overall covariate balance was better than that observed when stratifying patients according to IABP use (Table 1). However, such differences in covariates between IV-stratified groups are often reported in IV analyses[8, 15]. We also calculated the covariate imbalance using the Mahalanobis distance, which corrects for observed covariance among measured covariates. Stratification by the IV resulted in an 51% reduction in Mahalanobis distance, indicating a significant improvement in covariate balance. Adopting the framework proposed by Brookhart and Schneeweiss[13], when examining the difference in IABP use amongst various patient subsets stratified according to the IV, the strength of the IV was similar to that in the overall cohort across most observed variables, with the exception of 3 variables. The balance in the distribution of most observed variables provided reasonable evidence to infer that balance of unmeasured variables is likely to be improved by IV stratification. IABP was found to be associated with increased 3-year mortality, with an unadjusted mortality difference of 15.7% (95% CI: 8.4%, 30.0%, p<0.001) and absolute mortality difference of 11.5% (95% CI: 3.7%, 19.3%, p = 0.004). However, when performing an IV analysis, this association was lost and the IV–adjusted mortality difference was 16.7% (95% CI: -12.7%, 46.1%, p = 0.281).

**Table 3:** Examining the strength of centre-specific rate of IABP use as an instrumental variable.

	Baseline characteristics according to instrumental variable			IABP use within each subgroup according to instrumental variable			
	Centre IABP use			Centre IABP use		% difference in IABP use (95% CI)	
	Low (n = 243)	High (n = 457)	p	Low (n = 243)	High (n = 457)		
**Clinical factors**							
Age > 80 years	13.6	16.6	0.289	12.5	33.2	22.9	(12.5–33.2)
Female	26.3	25.4	0.783	14.3	29.1	21.7	(14.3–29.1)
PVD	9.1	5.5	0.080	-2.2	29.5	13.6	(-2.2–29.5)
Renal disease	7.2	14.7	0.008	20.2	47.3	34.2	(20,2–47.3)
Previous CVA	6.4	8.0	0.449	0.4	32.3	16.3	(0.4–32.3)
Previous MI	17.7	16.6	0.721	16.9	35.8	26.1	(16.9–35.8)
Previous revascularization	13.6	20.8	0.019	21.9	40.5	31.3	(21.9–40.5)
History of heart failure	18.0	15.1	0.340	9.8	31.6	21.0	(9.8–31.6)
Diabetes	22.2	26.0	0.288	15.8	32.5	24.2	(15.8–32.5)
Hypertension	51.6	48.4	0.436	18.5	29.7	24.1	(18.5–29.7)
Hypercholesterolemia	35.4	36.7	0.761	15.6	28.8	22.2	(15.6–28.8)
Smoking ^†^	38.4	21.7	<0.001	9.7	23.5	16.5	(9.7–23.5)
Severe LV (EF<30%)	10.1	28.9	<0.001	37.6	59.2	49.1	(37.6–59.2) *
Pulmonary disease	12.0	12.0	0.976	11.4	34.7	23.1	(11.4–34.7)
Gastrointestinal disease	9.2	10.6	0.582	18.3	42.9	30.3	(18.3–42.9)
Malignancy	6.9	6.2	0.735	7.8	39.8	23.8	(7.8–39.8)
**Coronary anatomy**							
LMS	8.6	9.2	0.796	27.1	56.6	42.9	(27.1–56.6)
LAD	76.1	78.0	0.570	20.2	29.2	24.6	(20.2–29.2)
proximal LAD	38.7	39.8	0.777	19.8	33	26.5	(19.8–33)
non-proximal LAD	56.0	66.6	0.006	22.7	32.8	27.8	(22.7–32.8)
LCx	51.0	61.5	0.007	23.1	33.6	28.5	(23.1–33.6)
RCA	67.5	70.3	0.438	18.7	27.9	23.3	(18.7–27.9)
Multivessel disease	67.1	72.7	0.117	21.6	39.9	26.3	(21.6–39.9)
**Procedural characteristics**							
Pre-procedural thrombolysis	28.0	7.4	<0.001	-7.8	9.8	1.0	(-7.8–9.8)
Radial access	41.6	14.4	<0.001	-3.2	4.5	-0.6	(-3.2–4.5)
GP 2b/3a inhibitor use	3.8	45.4	<0.001	30.5	46	38.3	(30.5–46.0) *
Thrombectomy	35.4	27.4	0.027	9.4	22.9	16.1	(9.4–22.9)
DES use	41.8	46.7	0.252	21	32.5	26.7	(21.0–32.5)
**Target vessel**							
LMS	4.9	7.5	0.199	28.2	63.6	47.8	(28.2–63.6)
LAD	47.3	48.6	0.754	22.3	40	28.3	(22.3–40.0)
proximal LAD	26.7	31.2	0.219	24.8	40.4	32.9	(24.8–40.4)
non-proximal LAD	28.4	36.0	0.041	26.7	40.2	33.5	(26.7–40.2)
LCx	20.2	30.8	0.003	27.2	43.3	35.4	(27.2–43.3)
RCA	43.6	43.7	0.977	14.3	25.2	19.7	(14.3–25.2)
Graft	1.2	1.1	0.873	-17.9	65.1	25.0	(-17.9–65.1)
Multivessel intervention	16.7	26.0	0.004	31.5	49.6	40.9	(31.5–49.6) *
				**IABP use as a % of the full cohort:**			
				4.4	27.7	23.3	(16.5–30)

Distribution of covariates between the groups and IABP use within each subgroup when stratified IV (expressed as %).

* indicates where variation in IABP use induced by the instrument for each covariate was larger or smaller than that observed in the overall cohort.

## Discussion

In patients presenting with STEMI and cardiogenic shock, the benefit of IABP therapy continues to be debated. Randomized studies have not shown a survival benefit with IABP therapy, whilst non-randomized studies have largely shown increased mortality. Non-randomized studies may be hampered with bias and confounding and despite traditional adjustment methods, there is always residual confounding, largely attributable to unmeasured confounders. Our study represents the first observational study in this field to systematically address the issue of measured and unmeasured confounding. The results of this study have shown that using traditional adjustment methods, IABP therapy is an independent predictor of increased mortality up to 3 years, largely in keeping with previous reported observational analyses[16–20]. However, when adjusting for measured and unmeasured confounders, this association was lost, and the results were in keeping with randomized trial data.

Pooled analyses of the studies with patients undergoing primary PCI demonstrate that IABP therapy is associated with increased mortality (30-day mortality difference = 6%, 95% CI, 3–10%, p<0.001)[6]. To date, there are 4 observational studies that have examined the benefit of IABP therapy in patients with cardiogenic shock in the primary PCI era[16–20]. The NRMI-2 study represents the earliest and largest of these (n = 8671) and found IABP therapy to be associated with increased in-hospital mortality, despite multivariable adjustment and using propensity score methods (OR = 1.27, 95% CI: 1.07–1.50)[18]. The observed detrimental effect of IABP therapy as an adjunct to primary PCI in STEMI with cardiogenic shock is contrary to the expectation that IABP might improve survival in these patients and the reasons for this remain speculative. A systemic inflammatory response to the device, as well the increase in access-site complications may be contributory[19]. However, the results of these observational analyses need to be interpreted with caution, as one cannot exclude the presence of confounders. Although the NRMI-2 study[18] adjusted for measured confounders it did not specifically address unmeasured confounders. Importantly, IABP therapy may have been given to the sicker patients, which would induce a severe bias towards poor outcomes in the IABP group.

The IABP-SHOCK II trial was a randomized evaluation of IABP therapy for acute myocardial infarction and cardiogenic shock in 600 patients, and represents the largest randomized body of evidence in this field[7]. IABP therapy had a neutral effect with no difference in 30-day mortality (RR = 0.96; 95% CI: 0.79–1.17, p = 0.69)[7]. Importantly, there was no divergence in the mortality curves, as was seen with the long-term results of the earlier IABP trials^1 [^^21^^]^, and the results were sustained up to 1 year (RR = 1.01; 95% CI: 0.86–1.18, p = 0.91)[22]. These data have challenged the notion that IABP therapy would improve outcomes by augmenting coronary perfusion. The lack of benefit may reflect the fact that the hemodynamic improvement with IABP and its effect on cardiac output is only modest with an absolute increase in cardiac output of 0.5 L/min[5], which might not be sufficient to reduce mortality. The insertion of IABP before coronary revascularization may improve procedural safety by improving left ventricular unloading[23], but data regarding this is somewhat conflicting[23, 24]. In the IABP SHOCK II trial, there was no mortality benefit in patients in whom the IABP was inserted before revascularization, as compared to after revascularization[7].

Similar to previous observational studies[16–20], the IABP population in our study was representative of the sicker and higher risk patient population. Using standard adjustment methods, we found IABP therapy was associated with increased mortality[16–18]. However, when adjusting for measured confounding using propensity-matched analyses, this association was lost. This loss of association with IABP therapy and mortality following propensity-matching has been reported in patients with STEMI[25]. However, a limitation with propensity-matched analyses, particularly when the treated group is small and with standard 1:1 matching, is that the overall study population analyzed is reduced, e.g. our propensity matched cohort of 278 patients represented 40% of the total study cohort. We addressed this with a 1:many matching algorithm, but despite this, IABP was still not associated with mortality. These results differ to those from the NRMI 2 study which demonstrated worse outcomes with IABP therapy despite adjustment using propensity score methods[18]. Whilst these differences may reflect unmeasured confounders, they may also be explained by the differences in the study cohorts. Compared to our study, the NRMI2 study cohort was a higher risk cohort exhibiting greater mortality rates. In the NRMI-2 study, patients were older; and comprised of both non-STEMI (63%) and STEMI (37%) patients compared to our study that exclusively had STEMI patients. The mortality of patients with non-STEMI and cardiogenic shock is significantly greater than those with STEMI and cardiogenic shock[26]. We also addressed unmeasured confounding using IV analyses, a recognized method for addressing treatment selection by unmeasured factors. These analyses confirmed findings from the propensity-matched cohorts. This study demonstrates how systematically addressing bias using propensity score and instrumental variable methods can reduce the gap between randomized trials and real world observational analyses.

Whether or not IABP therapy may be beneficial in select patient sub-groups remains to be determined, and the results from subgroup analyses of the IABP-SHOCK II trial were inconclusive[7]. Given the residual confounding with standard adjustment methods, we performed subgroup analyses using propensity-score adjusted models. Specifically, this demonstrated that IABP therapy was associated with increased long-term mortality in non-diabetic patients and those not undergoing multivessel intervention. Diabetic patients have greater coronary disease burden are more likely to have diffuse distal disease[27, 28]. Thus diabetic patients and particularly those undergoing multivessel intervention are more likely to have an increased ischemic burden, and the results from the subgroup analyses would suggest that these patients might benefit from IABP therapy. We observed a strong trend for worse outcomes in patients without renal disease. This is contrary to common perception that IABP therapy may compromise renal blood flow, and potentially compromise renal function in those with pre-existing renal disease, which may contribute to adverse outcomes. However, a possible explanation for our finding is that patients with renal disease are more likely to represent those with severe coronary disease and greater ischemic burden, who are more likely to derive benefit from IABP therapy. We also observed worse outcomes in those not receiving pre-procedural thrombolysis, suggesting possible benefit of IABP therapy in those undergoing rescue PCI following thrombolysis. This would be in keeping with findings from earlier studies in the thrombolysis era suggesting a benefit of IABP therapy following thrombolysis[6]. It is important to note that given the reduction in sample size in sub-group analyses, the power to detect differences is reduced. As such, the results of the subgroup analyses should be interpreted with caution. Whilst the presented subgroup analyses provide an interesting perspective, the findings should only be considered hypothesis generating. They must not distract us from the main finding of the study, where IABP had a neutral effect with respect to long-term mortality in patients undergoing PPCI for STEMI.

The main strength of this study is that it is the largest reported analysis examining IABP therapy exclusively in patients with STEMI and cardiogenic shock with the longest reported follow-up and addresses measured and unmeasured confounding. This study has all the limitations of a registry and all the potential bias associated with non-randomization, and despite our rigorous attempts to address confounding, residual confounding cannot be excluded. Data with regards to ventilation status, ischemic times, inotropic support, timing of IABP insertion (pre- or post revascularization) and peri-procedural hemodynamic data were not available. It has been shown that in the contemporary era, despite a reduction in ischemic times, including patients with cardiogenic shock, the mortality rates have remained the same. Thus the lack of ischemic times in this study is a less significant limitation. However, the use of an IV approach should address the presence of unmeasured variables. Nevertheless, the results of our study indicate that when adjusting for confounding, IABP therapy for patients with STEMI and cardiogenic shock undergoing PPCI is not associated with survival, which is in keeping with randomized trial data.

## Conclusions

In this observational analysis of patients with STEMI and cardiogenic shock undergoing PPCI, when adjusting for measured and unmeasured confounding, IABP therapy had a neutral effect and was not associated with long-term mortality. These findings differ to previously reported observational studies, but are in keeping with randomized trial data.
